# Anteromedial Impingement in Chronic Lateral Ankle Instability: A Comparison of MRI and Arthroscopic Findings

**DOI:** 10.7759/cureus.41982

**Published:** 2023-07-16

**Authors:** Don Koh, Darshana Chandrakumara, Charles Kon Kam King

**Affiliations:** 1 Orthopaedics, Changi General Hospital, Singapore, SGP; 2 Orthopaedic Surgery, Changi General Hospital, Singapore, SGP

**Keywords:** ankle mri, anteromedial impingement, arthroscopy, chronic ankle instability, atfl, ankle and foot

## Abstract

Introduction

Chronic lateral ankle instability (CLAI) is a known complication of ankle sprains, most commonly involving injury to the anterior talofibular ligament (ATFL). Growing evidence has shown an association between anteromedial (AM) impingement and CLAI. The purpose of this paper is to compare magnetic resonance imaging (MRI) with arthroscopic findings for the incidence of AM impingement in CLAI.

Methods

A retrospective study was performed by analyzing the radiological and operative reports of all patients who underwent an arthroscopic Broström-Gould procedure for CLAI between 2021 and 2022 at Changi General Hospital, Singapore. All patients who had a pre-operative MRI ankle scan performed and mention of the presence or absence of AM impingement in the operative notes were included in this study. Patients with concomitant fractures or systemic conditions affecting the same ankle were excluded.

Results

Ninety-seven patients were included in this study, 65 males and 32 females; 6.2% (6 of 97) of patients had a suggestion of AM impingement based on MRI findings, and 77.3% (75 of 97) of patients were noted to have AM impingement based on arthroscopic findings.

Conclusion

There is a high incidence of AM impingement associated with CLAI. AM impingement is often missed based on MRI findings. Arthroscopic Broström should be considered to address both issues of AM impingement and CLAI in the same setting.

## Introduction

The lateral ankle is stabilized by the anterior talofibular ligament (ATFL), the posterior talofibular ligament (PTFL), and the calcaneofibular ligament (CFL). The ATFL originates from the anterior surface of the distal fibula and inserts on the anterolateral corner of the talar articular facet, with its primary function to resist inversion in plantarflexion and anterolateral translation of the talus in the mortise [1]. The ATFL is the most commonly injured of the three lateral ligaments, owing to it being the shortest and weakest, and is involved in the majority of ankle inversion injuries [2]. Up to 20% of all acute ankle sprains eventually lead to chronic lateral ankle instability (CLAI) as a complication, with the ATFL likewise being the main ligament involved with this pathology [3]. CLAI itself is characterized by either functional or mechanical instability. Presenting complaints often include recurrent sprains or apprehension, resulting in activity limitation and decreased quality of life [4,5].

Ankle impingement syndrome is a condition characterized by pain and a restricted range of motion due to the entrapment of soft tissue lesions in the ankle joint. Ankle impingement syndromes can be further classified based on the anatomical region of the ankle affected [6]. Anteromedial (AM) impingement is caused by soft tissue lesions such as partially torn deep deltoid and thickened AM tibiotalar ligaments, located in the junction between the medial malleolus of the tibia and medial aspect of the talus [7]. During ankle dorsiflexion, impingement is caused by these lesions on the AM aspect of the talus resulting in the associated symptoms. Formation of osteophytes, chondral lesions, or impingement of tendons or other synovial structures has also been reported as a consequence of impingement.

The etiology of ankle impingement syndromes has not yet been fully elucidated, although trauma has been generally accepted to be the most likely cause [8]. Growing evidence has shown an association between ankle inversion injuries resulting in medial and lateral ligamentous injury with AM impingement [9]. Apart from the setting of a single major traumatic event, recurrent ankle sprains as with those with CLAI themselves have also been hypothesized to result in ankle impingement due to abnormal repetitive micromotion [10]. If left untreated, the symptoms of AM impingement such as pain and a reduced range of motion can lead to drastic limitations of daily activities and quality of life.

The initial approach to CLAI and AM impingement includes a thorough clinical history and physical examination and plain film radiographs. Further imaging with ultrasound (US) and magnetic resonance imaging (MRI) scans are often performed to aid in diagnosis [11].

This paper aims to report the incidence of AM impingement in CLAI as reported on MRI findings and compare them to findings noted during arthroscopy.

## Materials and methods

All patients who underwent a Broström-Gould (BG) procedure for CLAI between 2021 and 2022 at Changi General Hospital, Singapore by an experienced foot and ankle surgeon (CK) were identified for this study. Only patients who had a pre-operative MRI of the affected ankle performed and a record of the presence or absence of AM impingement in their operative notes were included. Patients with fractures or systemic conditions affecting the ankle were excluded. Finally, the MRI ankle reports and operative notes were retrospectively analyzed after receiving the IRB (Institutional Review Board) approval for this study.

The MRI ankle reports and operative notes were analyzed separately by the three authors. AM impingement was taken to be present if there was a report of soft tissue lesions present in or adjacent to the AM gutter. AM impingement was also taken to be present if it was reported on MRI as “suggestive of”, or “likely”. When the MRI reporting terms were ambiguous, the three authors convened and came to an agreement.

AM impingement was directly reported based on the statement of its presence or absence in the operative notes. AM impingement was reported as present if soft tissue lesions were noted to be present in the AM gutter. The ankle was also actively ranged with the AM gutter under direct arthroscopic vision and observed for any impingement.

## Results

Patients

The study included a total of 97 patients, 65 males and 32 females. The average age of the patients at the time of surgery was 34.4 years (range 17-66 years), and the average body mass index at the time of surgery was 30.1 kg/m^2^ (range: 18.1-40.2 kg/m^2^). Surgery was performed on 49 right and 48 left ankles. All 97 patients had the BG procedure performed arthroscopically. Table 1 provides a summary of the aforementioned information. 

**Table 1:** Patient demographics

Measurement	Value
No. of patients	97
Age (Yr)	34.4 ± 13.8 (range: 17-66 years)
Gender	Male 65 (67.0%)
	Female 32 (33.0%)
Body mass index (kg/m^2^)	30.1 ± 7.0 (range: 18.1 – 40.2 kg/m^2^)
Laterality	Right 49 (50.5%)
	Left 48 (49.5%)
Surgical approach	Arthroscopic 97 (100%)

Arthroscopic technique

The patient is positioned supine and placed under general or regional anesthesia followed by standard cleaning and draping protocols. A 30-degree 4mm arthroscope (LENS Integrated System, Smith & Nephew) is used with the standard AM and anterolateral portals. The medial gutter is identified and cleared of any soft tissue lesions if AM impingement is noted intraoperatively before proceeding with the BG repair.

AM impingement

**Figure 1:**
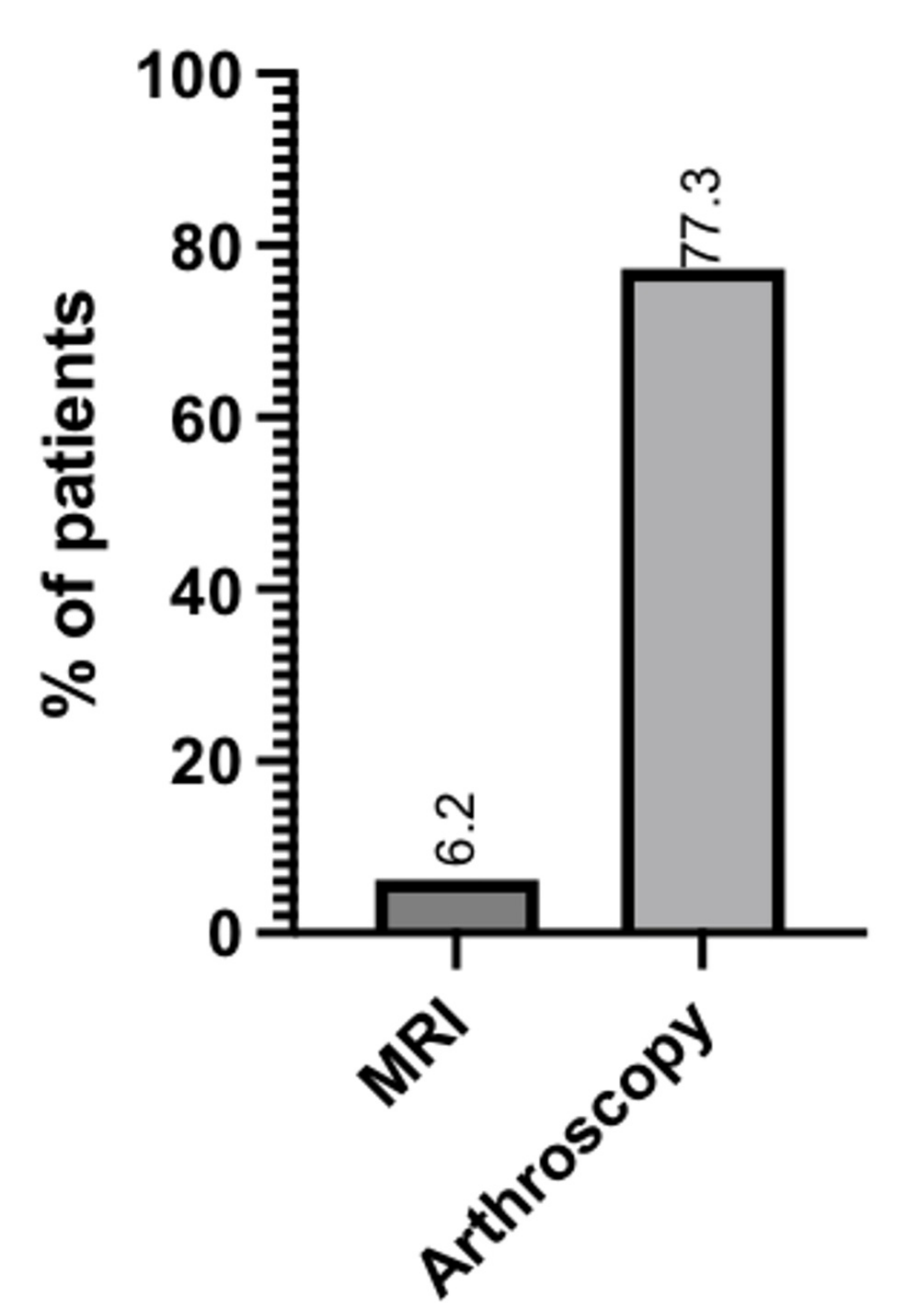
Percentage of patients with AM impingement reported based on MRI vs. arthroscopic findings AM: Anteromedial

6.2% (6 of 97) of patients had a suggestion of AM impingement based on MRI findings, and 77.3% (75 of 97) of patients had lesions noted in the junction between the medial malleolus of the tibia and the medial aspect of the talus consistent with AM impingement. The aforementioned information is displayed in Figure 1 and Table 2.

**Table 2:** Number of patients with AM impingement as noted on MRI reporting and during arthroscopy AM: Anteromedial

Modality	No. of patients with AM impingement
MRI Reporting	6
Arthroscopic findings	72

## Discussion

The ATFL is the primary ligament injured in both settings of acute ankle inversion injuries and subsequently, CLAI. AM impingement was seen to have a high incidence (77.3%) in association with ATFL injury (75 of 97 patients). The proposed mechanism for its development is chronic inflammation in the ATFL ligament, resulting in the formation of scar tissue which then forms a focus for impingement [12]. AM impingement was only detected in 6.2% (6 of 97) of patients based on MRI in our study. In accordance with existing knowledge, MRI has shown poorer sensitivities and specificities than clinical exams for AM impingement and is thus often missed [13].

As AM impingement is often implicated with ATFL injury, one consideration would be thus to address both issues in the same setting. Surgical treatment for CLAI is indicated for patients who have failed a trial of conservative management. ATFL primary repair has shown good outcomes since it was first described by Broström in 1966 [14]. The BG procedure and its subsequent variations involve anatomical repair of the ATFL and are now widely accepted as the gold standard treatment for CLAI. For AM impingement, surgical management involves debridement of the culprit soft tissue lesions.

Both the BG procedure and debridement for AM impingement can be performed via either open or arthroscopic approaches. Arthroscopic BG has shown good outcomes over open BG in terms of clinical outcomes and postoperative complications in various studies [15,16]. Likewise, for AM impingement, arthroscopic debridement has also shown good functional outcomes [17].

Advances in arthroscopic techniques have delivered comparative outcomes to open surgery for AM impingement, with even lower rates of complications [18]. A recent study by Yang et al. has also shown good clinical outcomes when both arthroscopic debridement for anterior ankle impingement and open modified Broström were performed together [19]. Although more technically challenging, an arthroscopic BG would confer the benefit of evaluating for and treating AM impingement in the same setting with reduces risk of complications compared to open surgery.

Limitations

Firstly, our study only allows us to state the association of AM impingement with CLAI in the South-East Asian population. Secondly, the indication for MRI ankle is to evaluate for ligamentous injury to the ATFL, hence possibly leading to under reporting of AM impingement by the respective radiologists as there was no highlight of that clinical concern. Thirdly, intraoperative findings of AM impingement were reported solely by a single surgeon. Intraobserver and interobserver reliability with regard to reporting the presence of AM impingement was not validated for the purposes of our study. 

While MRI is still the imaging modality of choice for AM impingement, further imaging studies are still required as sensitivities are still lacking compared to clinical examination in its diagnosis. Furthermore, the study can be further extrapolated to include a larger number of patients, as well as having the reporting radiologists and arthroscopic surgeons evaluated for their reliability.

## Conclusions

There is a high incidence of AM impingement associated with CLAI. AM impingement is often missed based on MRI findings. Arthroscopic Broström is a good approach which should be considered over open surgery, as it allows treatment of CLAI as well as evaluation and treatment of any concurrent AM impingement in the same setting. For those who already prefer the arthroscopic approach for BG, consideration should be taken to evaluate for AM impingement due to its high incidence in association with CLAI.
